# Cholera in Frankfort-On-The-Main

**Published:** 1855-12

**Authors:** G. Varrentrapp

**Affiliations:** Physician to the Hospital of the Holy Ghost, Frankfort-on-the Main.


### CHOLERA IN FRANKFORT-ON-THE-MAIN.

By G. Varrentrapp, M.D.,
Physician to the Hospital of the Holy Ghost, Frankfort-on-the Main.

In the different epidemics of cholera in Germany that took place after 1831, the south-western part of Germany never was seized. The northern part of Germany (particularly the kingdom of Prussia) suffered severely of that epidemic at repeated times; the south-eastern part likewise, Bohemia for instance, and Vienna itself. There was only one irruption in the south-western part (*i. e.* Bavaria, Wurtemburg, Baden, Hessen, Darmstadt, Nassau, Frankfort, and the southern part of the Saxon duchies); *i. e.*, from Tyrol the epidemic went to the village of Mittelwalde (village at the frontier of Bavaria and Tyrol), and from there to Munich. The cases of cholera at Munich were not very numerous at that first epidemic; the propagation from there went only to two places to Augsburg, where, if I am not wrong, two cases happened, and to Uffenheim (near Wurzburg in Franconia). Two Greeks had left Munich, during the time that cholera was in Munich; they were seized by cholera the next day at Uffenheim: one of them died, and two or three persons having been in direct communication with them at Uffenheim, were seized likewise and died. Uffenheim is at a great distance from Munich, I think nearly a hundred English miles. From the northern part, Cologne and Hesse Cassel were the next places of south-western Germany, where cases of cholera were observed. At Cassel the epidemic was of short duration; the number of cases was not very large, but the proportion of deaths was considerable. These are the principal points in question relating to the first epidemics that I recollect. As to further details, they may easily be obtained by addressing to any physician at Cassel, Cologne, or Munich. At Frankfort, &c., not one single case was observed.

In 1849, after many years without cholera, this epidemic again reached Germany, and this time cholera came to us and to our neighbourhood likewise. Cholera had been in summer time in the northern part of Germany, in Prussia, Mecklenbourg, Holstein, &c. The cholera came from the east to the west through the northern Germany, and when it had reached the Rhine it turned southward. It took a very particular course, and presented one particularity in this course, to which, as far as I am aware, nobody has paid attention, though it be very interesting. I said the cholera came gradually up the Rhine. There is nothing remarkable in this way of spreading, the Rhine being, as every body knows, one of the most frequented lines of communication that can exist. There are large and wealthy commercial towns, and a rich agricultural population along the Rhine, on both sides of the river good public roads, and the great communication by steamers on the Rhine itself. Yet what I am to communicate now, will prove that the cholera did not take this way because there was a constant intercourse and communication of men, but the cholera followed merely the valley of the Rhine. There must have been at that time very decisive influences either of a local telluric nature or something similar, which induced the disease to take this way, and which prevented it from deviating either to the right or to the left, even when there was in one of these directions a communication of men quite as considerable as in any part along the Rhine itself. The astonishing part is this:—

1. The cholera has been in Coblenz, but it did not go up the Lahn to Ems, though this is less than seven English miles from Coblenz, and though there is in summer time a considerable intercourse between Coblenz and Ems.

2. The cholera has been at Bingen, but it did not follow the river Nahe and did not reach Creuznach, being at a distance of from eight to ten English miles, and being in lively communication with Bingen.

3. The cholera has been at Mainz, but it did not come to Wisbaden, at about four miles distance from Mainz; nor did it really attack Frankfort, though we had a few cases, of which I will speak very soon. Mainz has, with no other places, so much intercourse as with Frankfort and Wisbaden.

4. The cholera has been at Mannheim, but it did not go up the Neckar to Heidelberg (about ten miles distance), though Heidelberg has with no other place so much communication as with Mannheim.

These data are very strange. The cholera evidently followed the valley of the Rhine; it did not propagate to the country on either side, and it did not follow neither merely the course of the waters; at least it did not go up any of the secondary rivers just mentioned, though along these rivers there is a great movement of the population. We must say, I think, that the cholera in its propagation from Cologne did not follow the great lines of communication as far as this was a communication by land, but that it followed the line of communication by water merely as far as this related to the principal valley of the Rhine, and that it did not enter any lateral valley.

At Mannheim (with 23,410 inhabitants) there were observed 710 cases of cholera, 374 of which were fatal. The first cases happened the 24th August, 1849; the epidemic diminished from the 7th October, and it was nearly over the 26th October; from the 27th October to 18th November there happened only 17 cases (13 of which were fatal).*

At Mainz (a fortress with nearly 80,000 inhabitants), the number of cases of cholera was, I think, between 300 and 400. At Mainz there work generally (particularly in the building professions) a great number of the natives of the neighbouring villages in the duchy of Nassau. They generally return to their homes on Saturday evening, and go to their work at Mainz on Monday morning again. Several of these individuals (it may have been a dozen perhaps) caught the cholera at Mainz; feeling ill they went home; here they showed all the symptoms of cholera: the majority of them recovered. Yet there being no local disposition for any spreading of the disease, the cholera did not attack one regular inhabitant of these villages. There was only one single exception of it. A steersman being

* For further statistical details, see the Report of Dr. Frey in Wunderlich's *Archiv. für Physiologische Heilkunde*, Bd. IX, pp. 270 297.

at Mainz, felt the first symptoms of cholera; he hurried home to Höchst, between Frankfort and Mainz (fifteen English miles from Mainz, five English miles from Frankfort); he got a severe attack, but he recovered. His wife had taken care of him during his disease; she got the cholera: her father took care of her during her disease, he fell ill likewise. One of them or both died. These were the three only cases observed at Höchst.

In Frankfort itself, we had, in September, October, and November 1849, seven cases of cholera. They all, with the exception of the first one, were under my medical treatment in the hospital of the Holy Ghost.

1. In the last days of August, or in the first days of September 1849, a wine merchant of Frankfort, immediately after his departure from Hamburg, got the first symptoms of cholera; yet he could reach Frankfort. After a few days' duration of the disease he died.

2. A sculptor travelled on the 19th of September from Cologne to Bingen, where his child had been seized by cholera. The child recovered; the family would then continue the journey to Frankfort. On the 11th of October, in the evening, and on the 12th, he took an excess in sweet wine (wine before fermentation), sour beer, and brandy and saucisse. In the night of the 13th, he was taken by cholera; in the middle of the day he was brought to the hospital—sixteen hours afterwards. Twenty hours after the first beginning, he died.

3. On the 10th of October, a young labourer came to our hospital. He had been in the hospital at Mannheim for hypochondriac bowel complaints for three weeks (the autopsy showed chronic dysenteric process), during which time many cholera patients had been in the same wards. On the 8th he left the hospital, and travelled to Frankfort. This same evening, and the next day, he drank much sweet cider (before fermentation). In the night of the 9th and 10th Oct., he was seized by cholera; he died on the 13th, seventy-three hours after his reception in the hospital, and eighty-five hours after the beginning of the disease.

4. The 1st November, a sailor came to us from Coblenz, who had slept on the night of the 30th and 31st of Oct. at Mainz. On the 31st, he arrived at Frankfort, and took the cholera. He recovered soon.

The cases 2, 3, and 4, were kept in a separate room, and in a separate story where there were no other patients.

5. On the 15th of November, a tailor came to our hospital, who had not left Frankfort for the last two months, and who had not been in contact with any cholera patient as far as he knew. On the 11th of November he had taken much young wine, in the beginning of the stage of fermentation. He had aqueous diarrhœa after it; and cholera in the night of the 14th and the 15th. He was better on the 17th, and was discharged on the 24th November. He had been treated alone in a small ward, which, besides the door going to the corridor, had a door of communication to the principal ward, with eleven beds. This door was opened at several times, and one nurse passed from one to the other of these wards.

6. The 19th November, in the afternoon, a patient, lying in the principal ward (just opposite to the door of communication), with chronic bronchitis, and who had not left the bed during several days, for a slight ulceration at the umbilicus, suddenly and vehemently was seized by cholera; on the 22nd, the real cholera symptoms were gone; but signs of cholera-typhus, or uræmia with otorrhœa commenced; on the 12th of December, sopor; on the 13th he died. Caries of the pars petrosa, ossis temporum, and a small abscess in the cerebellum quite near to the carious part of the pars petrosa, were found in this case.

7. In the second bed from this patient, a shoemaker was lying for endocarditis rheumatica; his disease had been thought to be a pericarditis, and he had been treated accordingly. After several days there was *no* symptom of disease left, but that shown by auscultation; for this reason he was engaged to remain in the hospital. He was much frightened by the first case of cholera, and still more by the second. On the 23rd of November, late in the evening, he was seized by cholera, and died next morning, twelve hours after the beginning of the disease.

These seven cases have been related by my assistant-physician of that time, Dr. de Neufville, in *Wunderlich's Archiv,* vol. ix, p. 456, *et seq.*

For several years after this we had no cases of cholera, till in 1854 cholera made its first real irruption in Frankfort. In the middle of August, a tradesman arrived here from Munich, where, at that time, cholera had made some severe ravages. Immediately after his arrival he got a mild attack of cholera; two days afterwards he was in full convalescence. In the night of the 29th and 30th August I was called to see, in consultation, a man (called Bader) in his best years, and of a vigorous constitution (a messenger in a house of commerce), who had taken, at eight o'clock in the evening, his supper in good health, when, short before midnight, he was seized by cholera in a very violent form, so that he died in the afternoon of the 30th. In the morning, one of his servants (he had two female servants to help his wife, who was a laundress) was taken by cholera, the other in the afternoon, at which time I had both transported to my hospital, where one recovered after a few days, and the other very slowly, after having undergone a typhoid reaction. One of them had had three days before, and the other one day previously, very distinct premonitory symptoms (diarrhœa and vomiting) of the disease, without saying a word of it, so that they had been ill *before* Mr. Bader. During the day (30th August) one of the children of Mr. Bader was taken with the disease and died in the night, so that on the following morning we sent Mrs. Bader with another child, who meanwhile had been taken by cholera likewise, from her narrow lodging to her native place (a village at three miles' distance from Frankfort). The child recovered; but the sister of Mrs. Bader, who had taken care of the child, was taken severely by cholera, but recovered. This case was the only case in this village, and the only case in all the villages in the neighbourhood of Frankfort last year. I made haste to learn from what cause cholera had arisen so suddenly, in the midst of our town; at last, I learnt that the shirts and other pieces of linen, of which the tradesman of Munich (first mentioned) had made use during his disease, had been given to Mrs. Bader to be washed, and that those two female servants had really washed them. Whether this was indeed the mode of contagion, was very uncertain to my mind, till the next case happened. A Jew merchant was taken by cholera several days after these first cases, with his child. The child died; the father recovered. The clothes, shirts, linen, etc., were sent to a laundress living in a very distant part of the town. A few days afterwards this laundress was seized with cholera, and died. Her children, living again in another part of the town, divided her clothes and linen among themselves, and took the linen of the Jew, in order to send it washed to him. They were likewise seized.

These cases, a few other similar ones in this epidemic, and the two last cases (6 and 7) in the epidemic of 1849, have convinced us physicians of this town, even those who formerly had been absolutely anticontagionists, that though contagion is perhaps not the principal mode of propagating this disease, yet there exist undeniable cases of contagion, and that it seems that the excrementa, or the linen, and clothes particularly, when polluted, are capable of propagating the disease. I have since made arrangements that in all such cases all linen (shirts, sheets of bed, etc.) are immediately put aside in a concentrated lye for a few days before being given to be washed.

In several cases that happened during the next months, errors in diet seemed to be the cause. In several instances, the disease was ascribed to the use of sweet cider in larger or smaller quantity. At all events, we should not suppose in our town, where a very large quantity of cider is drunk (100,000 to 150,000 hundredweights of apples being used for cider in a year), that cider could be of any use in cholera, or even in choleraic times; assuredly, of all drinks cider would be the first to be forbidden with us, in the same way as melons were forbidden in France, etc., or are forbidden in the time of epidemics of cholera.

I cannot tell you at this time the exact number of cholera patients we had during last autumn in Frankfort. The epidemic began in the end of August 1854 and lasted to October; I think we had forty or fifty cases. At nearly the same time, cholera appeared for the first time at Darmstadt (fifteen English miles from Frankfort; the first case there was the father of a student returning from Munich), and to Hanau (ten English miles from Frankfort). But the number of the cases was still smaller there than at Frankfort.*

* The above communication is the substance of a letter containing answers to the Cholera Queries issued by the Epidemiological Society. The remarks of the author specially refer to the first, second, third, and fourth queries; but they incidentally answer, in the negative, a query (No. 8) regarding the prophylactic properties of cider. [Editor of the Journal of Public Health.]

